# Crystal structure of bis­(1-mesityl-1*H*-imidazole-κ*N* ^3^)di­phenyl­boron tri­fluoro­methane­sulfonate

**DOI:** 10.1107/S2056989020005058

**Published:** 2020-04-21

**Authors:** Aniffa Kouton, Yafei Gao, Veronica Carta

**Affiliations:** a Department of Chemistry, Indiana University, 800 E Kirkwood Street, 47405, Bloomington (IN), USA

**Keywords:** crystal structure, weak inter­actions, bulky ligand

## Abstract

The solid-state structure of bis­(1-mesityl-1*H*-imidazole-κ*N* ^3^)di­phenyl­boron tri­fluoro­methane­sulfonate, C_36_H_38_BN_4_^+^·CF_3_SO_3_^−^ or (**Ph_2_B(MesIm)_2_OTf**), is reported. Bis(1-mesityl-1*H*-imidazole-κ*N* ^3^)di­phenyl­boron (**Ph_2_B(MesIm)_2_^+^**) is a bulky ligand that crystallizes in the ortho­rhom­bic space group *Pbcn*. The asymmetric unit contains one **Ph_2_B(MesIm)_2_^+^** cationic ligand and one tri­fluoro­methane­sulfonate anion that balances the positive charge of the ligand. The tetra­hedral geometry around the boron center is distorted as a result of the steric bulk of the phenyl groups. Weak inter­actions, such as π–π stacking are present in the crystal structure.

## Abstract 2

In this manuscript, we report the the crystal structure of di­phenyl­bis­(mesityl­imidazole)­borane tri­fluoro­methane­sulfonate. Weak inter­actions, such as π–π stacking are present in the structure.

## Chemical context   

**Figure 1:**
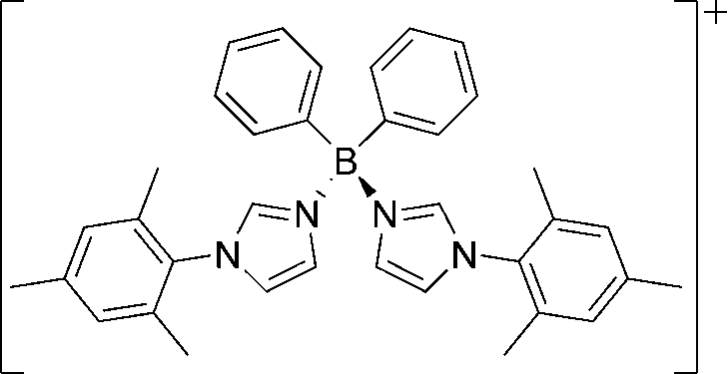
Chemical structure of di­phenyldi(mesityl­imidazole)­borane **Ph_2_B(MesIm)_2_^+^**.

**Figure 2:**
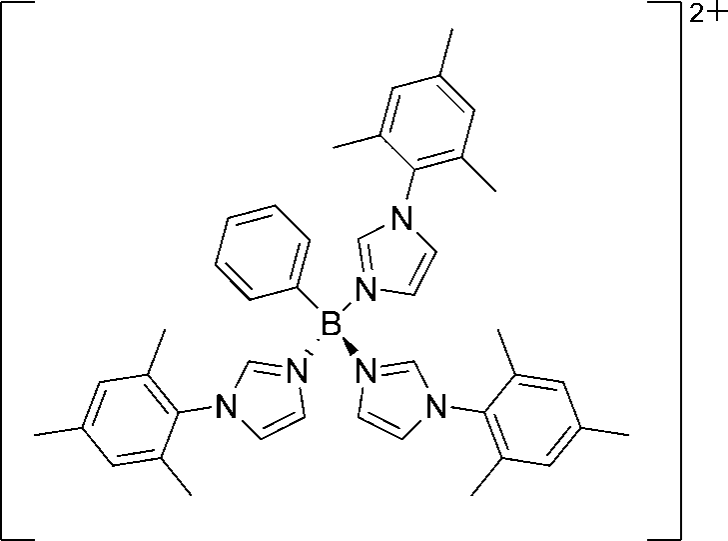
Chemical structure of phenyl­tris­(mesityl­imidazole)­borane **PhB(MesIm)_3_^2+^**.

**Ph_2_B(MesIm)_2_^+^** (Fig. 1 ▸) can undergo C—H activation on the imidazole functionalities, generating a bi(carbene)borate ligand, which can coordinate to a metal center with two carbenes. The ligand is bulky and has strong σ-donor character. For this reason, it can be used to stabilize a metal center. Similar bulky ligands, such as tris­(mesityl­imidazole)­phenyl­borane, **PhB(MesIm)_3_** (Fig. 2 ▸) have been used to synthesize iron nitride complexes (Smith & Subedi, 2012 ▸), which have shown promising applications in catalysis (Scepaniak *et al.*, 2009 ▸) and in the production of ammonia both in biological and in industrial processes (Smith & Subedi, 2012 ▸). The threefold symmetry and the bulk of **[PhB(MesIm)_3_]^2+^** ligand are key to stabilizing iron–nitro­gen multiple bonds and isolate the terminal iron nitride complexes (Smith & Subedi, 2012 ▸).
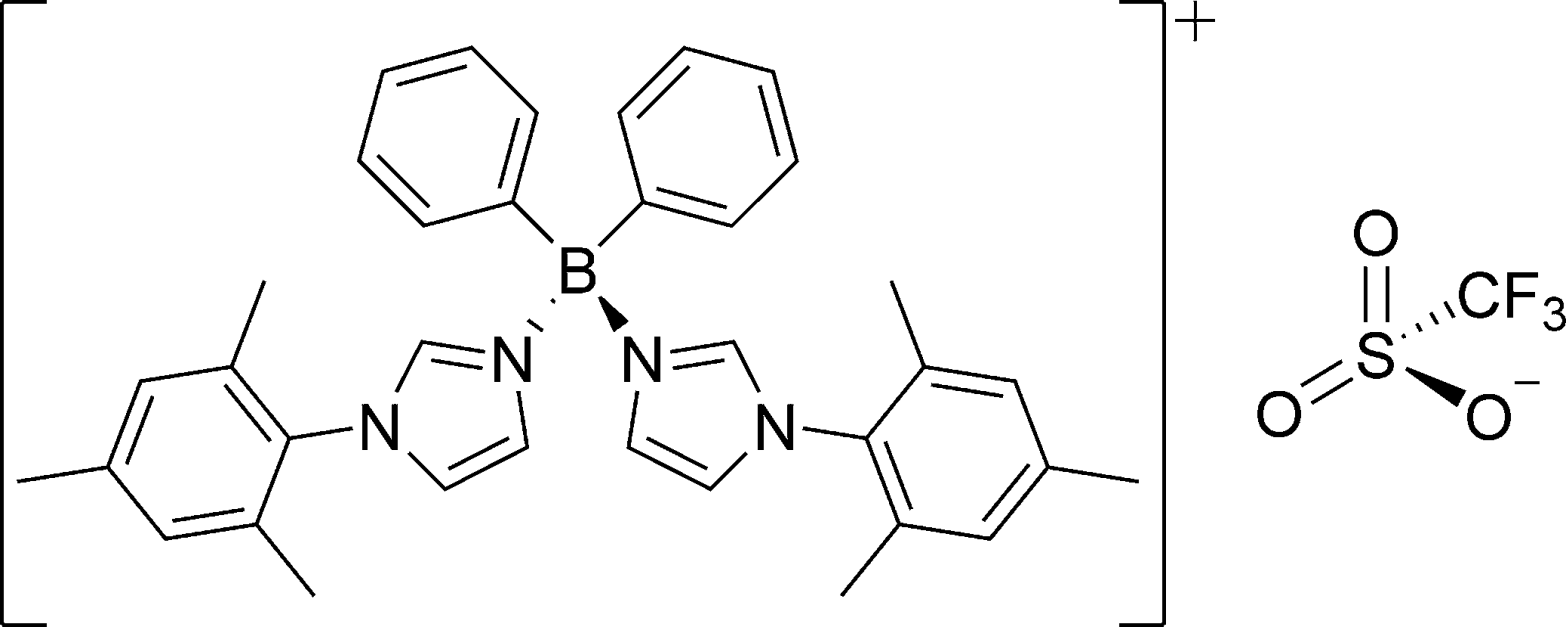


In this paper, we discuss the synthesis and crystal structure of **Ph_2_B(MesIm)_2_OTf**, which can potentially be used to synthesize low-coordinate metal complexes for small-mol­ecule activation and catalysis. The synthesis of **Ph_2_B(MesIm)_2_OTf** started from the reaction of 1 eq. of Ph_2_BCl with 2 eq. of 1-mesityl-1*H*-imidazole. The product was further reacted with 1 eq. of tri­methyl­silyl tri­fluoro­methane­sulfonate (TMSOTf) to yield the title compound.

## Structural commentary   

**Figure 3:**
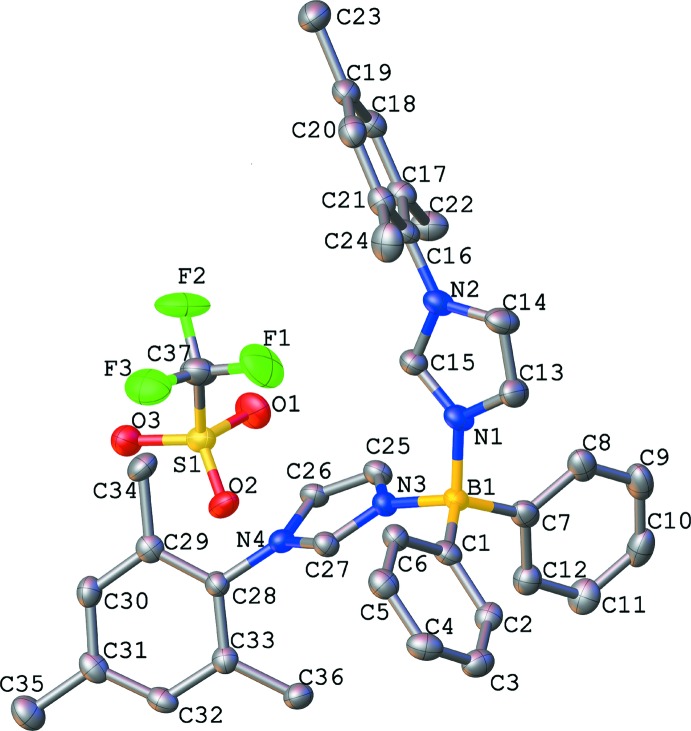
Mol­ecular structure of **Ph_2_B(MesIm)_2_OTf** with atom labels. Displacement ellipsoids are shown at the 50% probability level. Hydrogen atoms are omitted for clarity.

The title compound crystallizes in the ortho­rhom­bic space group *Pbcn*. The asymmetric unit consists of one **Ph_2_B(MesIm)_2_^+^** ligand and one triflate anion that balances the total positive charge of **Ph_2_B(MesIm)_2_^+^** (Fig. 3 ▸).

**Figure 4:**
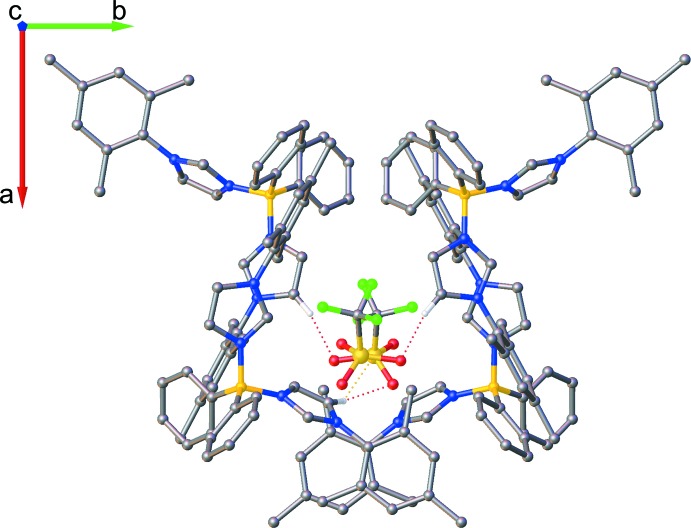
Partial packing diagram of **Ph_2_B(MesIm)_2_OTf** along the *c* axis. Hydrogen atoms are omitted for clarity. Dotteded lines indicate the weak inter­molecular inter­actions between tri­fluoro­methane­sulfonate and di­phenyldi(mesityl­imidazole)­borane.

The boron atom has tetra­hedral geometry. As a result of the steric repulsion of the phenyl groups, the angle between the boron and the two phenyl groups (C1—B1—C7) is 116.7 (3)° and larger than the typical tetra­hedral angle (109°), whereas the angle between the imidazole moieties and the boron center (N1—B1–N3) is smaller at 105.8 (3)°. The remaining two angles are 107.4 (3) and 109.3 (3)°. The bulky mesityl groups point away from each other, creating a pocket in which the triflate mol­ecule is located (Fig. 4 ▸). The dihedral angles between the imidazole and mesityl mean planes are 63.1 (2)° for N1/N2/C13–C15 and C16—C21, and 67.85 (17)° for N3/N4/C25–C27 and C28–C33. The dihedral angle between the mean planes defined by the phenyl rings on the boron atom (C1–C6 and C7–C12) is 58.28 (19)°.

## Supra­molecular features   

**Figure 5:**
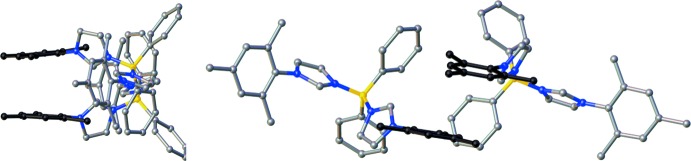
π–π stacking in the crystal structure of **Ph_2_B(MesIm)_2_^+^** between the mesityl ring C28–C33 and its neighboring symmetry-equivalent moiety. The rings involved in π–π stacking are represented in black.

Although no classical hydrogen bonds were found in the structure, weak inter­molecular inter­actions between the triflate anion and the protons on the imidazole groups are present (Table 1 ▸). The triflate anion also inter­acts weakly with one of the imidazole rings (N3/N4/C25–C27) through one oxygen atom (O1), with a centroid–oxygen distance of 3.529 (3) Å. Additional weak inter­actions, namely π–π stacking, are present in the packing for one of the mesityl groups (C28–C36), with a perpendicular distance of 3.5727 (13) Å between the mesityl ring (C28–C33) and the least-squares mean plane of a neighboring symmetry-equivalent moiety (Fig. 5 ▸). The centroid–centroid distance between the two mesityl rings is 3.947 (2) Å and the slippage between the two π-rings is 1.677 Å. The dihedral angle between the two mesityl mean planes is 7.58 (15)°. The second mesityl ring (C16–C24) is not involved in π–π stacking inter­actions, with the closest aromatic rings, C1–C6 and C7–C12, at centroid–centroid distances of 5.710 (2) and 5.139 (3) Å, respectively, and with mean-plane dihedral angles of 16.31 (19) and 49.8 (2)°, respectively. The two mesityl groups are almost perpendicular, subtending a dihedral angle of 88.39 (17)°.

## Database survey   

A survey of the Cambridge Structural Database (CSD Version 5.41, 2020.0 CSD Release; Groom *et al.*, 2016 ▸) was undertaken for structures related to **Ph_2_B(MesIm)_2_OTf**. One example is the structure of (3-butyl­imidazole)­tri­phenyl­boron [**Ph_3_B(3-ButIm)**; refcode OFAFIK; Stenzel *et al.*, 2002 ▸), a neutral mol­ecule with an additional phenyl ring instead of an imidazole group (three phenyl rings) and with an alkyl chain instead of the mesityl moiety. **Ph_3_B(3-ButIm)** crystallizes in the space group *P* 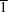, and has a very different crystal packing from **Ph_2_B(MesIm)_2_OTf**. However, the two mol­ecules have a similar geometry around the boron atom, with the tetra­hedral angles around the boron atom impacted by the bulky phenyl groups. The C—B—C angles involving phenyl moieties range between 108 and 114°, while the angles between imidazole and phenyl moieties are accordingly smaller (C—B—N angles of about 104–109°). **Ph_3_B(3-ButIm)** shows C—H⋯π inter­actions from the imidazole hydrogen to the phenyl ring. These inter­actions are not present in **Ph_2_B(MesIm)_2_OTf**, where the imidazole inter­acts only weakly with the triflate oxygen atoms. Another similar example is phenyl­imidazole tri­phenyl­borane [**Ph_3_B(PhIm)**; ACIPEH; Kiviniemi *et al.*, 2001 ▸]. **Ph_3_B(PhIm**) is a neutral mol­ecule with three phenyl rings on the boron atom and one phenyl ring on the imidazole functionality. **Ph_3_B(PhIm**) crystallizes in the monoclinic space group *C*2/*c* and again has a different crystal packing from **Ph_2_B(MesIm)_2_OTf**, characterized by chains that are stabilized by weak π–π stacking inter­actions between the phenyl groups on the imidazole.

The CSD search also revealed one di­phenyl­bis­(ada­man­tyl­imidazole)­borane chloride salt, **Ph_2_B(AdIm)_2_Cl** (CAX­MAS; Xiong *et al.*, 2017 ▸). In this compound, the imidazole functionalities are bound to adamantyl groups and the tetra­hedral boron atom is bound to two toluene and two imidazole groups. The protons on the imidazole groups inter­act *via* hydrogen bonds with the chloride anion, which is located in a pocket between the two bulky adamantyl groups, similar to that observed for the triflate anion in **Ph_2_B(MesIm)_2_OTf**. The crystal packing shows weak inter­molecular C—H⋯π inter­actions between the methyl group on the toluene functionality and the aromatic ring on the neighboring toluene. Despite some similarities with the title compound, **Ph_2_B(AdIm)_2_Cl** crystallizes in the space group *C*2/*c* and has a different crystal packing structure.

Few boron dimers with bridging imidazole groups were found in the CSD. One example is **[Ph** ***_2_*** **B(3-BuIm)]_2_** (FULPAE; Arrowsmith *et al.*, 2009 ▸), which crystallizes in the space group *C*2/*c*. In this boron dimer, the two tetra­hedral boron centers are bridged by two 3-butyl­imidazole groups and each boron atom is bound to two phenyl groups. A second example of a boron dimer is **[Ph** ***_2_*** **B(3-BuIm)]_2_** (PONLOW; Su *et al.*, 2019 ▸), space group *P*2_1_/*n*. In this compound one boron atom is bound to two phenyl groups and the second boron atom is bound to one chloride and one hydrogen atom. The boron atoms are bridged by two di­phenyl­mesityl­imidazole groups.

## Synthesis and crystallization   

**Figure 6:**
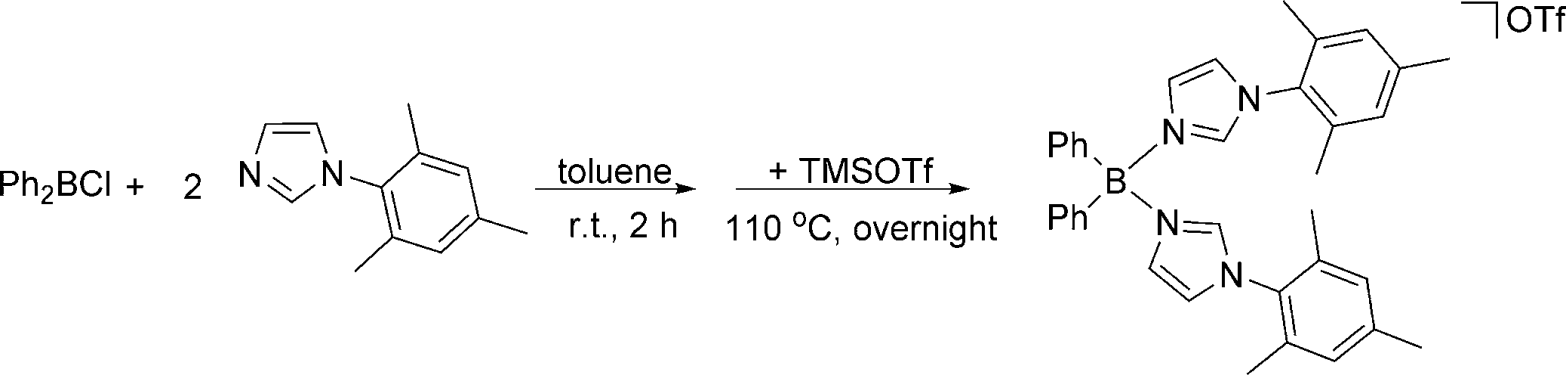
Reaction for the synthesis of **Ph_2_B(MesIm)_2_OTf.** Ph_2_BCl (1 equiv.), 1-mesityl-1*H*-imidazole (2 equiv.) were stirred in toluene at room temperature for 2 h. TMS OTf (1 equiv.) was then added and the mixture was further stirred at 383 K overnight.

The synthesis of **Ph_2_B(MesIm)_2_OTf** is shown in Fig. 6 ▸. A 25 mL flask was charged with Ph_2_BCl (914 mg, 4.5 mmol), 1-mesityl-1*H*-imidazole (1.7 g, 9 mmol) and toluene (10 mL). The mixture was stirred at room temperature for 2 h. During the course of the reaction, a white precipitate formed. Then TMS OTf (1.0 g, 4.5 mmol) was added as a brown liquid. The mixture was further stirred at 383 K overnight. The toluene was evaporated under vacuum, affording a white residue that was washed with Et_2_O (3 × 10 mL) to obtain **Ph_2_B(MesIm)_2_OTf** as a white powder (2.6 g, 79% yield). Single crystals suitable for X-ray diffraction were grown by vapor diffusion using diethyl ether and DCM. ^1^H NMR (400 MHz, CDCl_3_, 298 K): *δ* (ppm) 7.81 (*s*, 2H), 7.47 (*s*, 2H), 7.35 (*s*, 2H), 7.24–7.27 (*m*, 6H), 7.10 (*d*, *J* = 8.0 Hz, 4H), 6.93 (*s*, 4H), 2.25 (*s*, 6H), 1.99 (*s*, 12H). ^13^C NMR (101 MHz, CDCl_3_, 298 K): *δ* (ppm) 104.92 (*s*), 137.99 (*s*), 134.31 (*s*), 133.06 (*s*), 131.05 (*s*), 129.66 (*s*), 128.99 (*s*), 128.28 (*s*), 127.85 (*s*), 126.45 (s), 124.23 (*s*), 21.03 (*s*), 17.38 (*s*).

## Refinement   

Crystal data, data collection and structure refinement details are summarized in Table 2 ▸. The hydrogen atoms were placed in ideal positions and refined as riding atoms with relative isotropic displacement parameters [*U*_iso_(H) = 1.2 or 1.5 × *U*_eq_(parent atom)].

## Supplementary Material

Crystal structure: contains datablock(s) I. DOI: 10.1107/S2056989020005058/jj2222sup1.cif

Structure factors: contains datablock(s) I. DOI: 10.1107/S2056989020005058/jj2222Isup3.hkl

Supporting information file. DOI: 10.1107/S2056989020005058/jj2222Isup4.cdx

CCDC reference: 1983246

Additional supporting information:  crystallographic information; 3D view; checkCIF report

## Figures and Tables

**Table 1:** Weak inter­molecular inter­actions (Å, °) between the tri­fluoro­methane­sulfonate anion and the imidazole moieties in Ph_2_B(MesIm)_2_^+^

	Distance	Angle
C26⋯S1^i^	3.793 (4)	149.4
C26⋯O2^i^	3.264 (5)	160.0
C15⋯O1	3.132 (5)	118.7
C14⋯O3^ii^	3.242 (5)	159.4
N3—C27⋯O1	3.529 (3)	125.0

Symmetry codes: (i) *x*, −*y* + 1, *z* − 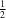; (ii) −*x* + 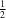, *y* + 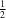, *z*.

**Table 2:** Experimental details

Crystal data	
Chemical formula	C_36_H_38_BN_4_^+^·CF_3_O_3_S^−^
*M*_r_	686.58
Crystal system, space group	Orthorhombic, *P* *b* *c* *n*
Temperature (K)	100
*a*, *b*, *c* (Å)	28.661 (4), 15.979 (3), 15.352 (3)
*V* (Å^3^)	7031 (2)
*Z*	8
Radiation type	Mo *K*α
μ (mm^−1^)	0.15
Crystal size (mm)	0.20 × 0.10 × 0.05

Data collection	
Diffractometer	Bruker Venture D8
Absorption correction	Multi-scan (*SADABS*; Bruker, 2016 ▸)
*T*_min_, *T*_max_	0.620, 0.746
No. of measured, independent and observed [*I* > 2σ(*I*)] reflections	55395, 8077, 4090
*R*_int_	0.207
(sin θ/λ)_max_ (Å^−1^)	0.650

Refinement	
*R*[*F* ^2^ > 2σ(*F* ^2^)], *wR*(*F* ^2^), *S*	0.084, 0.225, 1.01
No. of reflections	8077
No. of parameters	448
H-atom treatment	H-atom parameters constrained
Δρ_max_, Δρ_min_ (e Å^−3^)	0.29, −0.56

Computer programs: *APEX3* and *SAINT* (Bruker, 2016 ▸), *SHELXT* (Sheldrick, 2015*a* ▸), *SHELXL* (Sheldrick, 2015*b* ▸) and *OLEX2* (Dolomanov *et al.*, 2009 ▸).

## supplementary crystallographic information

### Crystal data

**Table:**

C_36_H_38_BN_4_^+^·CF_3_O_3_S^−^	*D*_x_ = 1.297 Mg m^−^^3^
*M**_r_* = 686.58	Mo *K*α radiation, λ = 0.71073 Å
Orthorhombic, *P**b**c**n*	Cell parameters from 783 reflections
*a* = 28.661 (4) Å	θ = 2.7–22.0°
*b* = 15.979 (3) Å	µ = 0.15 mm^−^^1^
*c* = 15.352 (3) Å	*T* = 100 K
*V* = 7031 (2) Å^3^	Plate, clear colourless
*Z* = 8	0.20 × 0.10 × 0.05 mm
*F*(000) = 2880	

### Data collection

**Table:**

Bruker Venture D8 diffractometer	4090 reflections with *I* > 2σ(*I*)
φ and ω scans	*R*_int_ = 0.207
Absorption correction: multi-scan (SADABS; Bruker, 2016)	θ_max_ = 27.5°, θ_min_ = 2.0°
*T*_min_ = 0.620, *T*_max_ = 0.746	*h* = −37→37
55395 measured reflections	*k* = −20→17
8077 independent reflections	*l* = −16→19

### Refinement

**Table:**

Refinement on *F*^2^	Primary atom site location: dual
Least-squares matrix: full	Hydrogen site location: inferred from neighbouring sites
*R*[*F*^2^ > 2σ(*F*^2^)] = 0.084	H-atom parameters constrained
*wR*(*F*^2^) = 0.225	*w* = 1/[σ^2^(*F*_o_^2^) + (0.086*P*)^2^] where *P* = (*F*_o_^2^ + 2*F*_c_^2^)/3
*S* = 1.01	(Δ/σ)_max_ < 0.001
8077 reflections	Δρ_max_ = 0.29 e Å^−^^3^
448 parameters	Δρ_min_ = −0.56 e Å^−^^3^
0 restraints	

### Special details

**Table:**

Geometry. All esds (except the esd in the dihedral angle between two l.s. planes) are estimated using the full covariance matrix. The cell esds are taken into account individually in the estimation of esds in distances, angles and torsion angles; correlations between esds in cell parameters are only used when they are defined by crystal symmetry. An approximate (isotropic) treatment of cell esds is used for estimating esds involving l.s. planes.
Refinement. A colorless crystal (plate, approximate dimensions 0.20 × 0.10 × 0.05 mm^3^) was placed onto the tip of a MiTeGen pin and mounted on a Bruker Venture D8 diffractometer equipped with a Photon II detector at 100.0 K. The data collection was carried out using Mo Kα radiation (λ = 0.71073 Å, graphite monochromator) with a frame time of 0.5 seconds and a detector distance of 50 mm. Complete data to a resolution of 0.77 Å with a redundancy of 4 were collected. The frames were integrated with the Bruker software package SAINT using a narrow-frame algorithm (Bruker, 2016) to a resolution of 0.77 Å.The space group P*bcn* was determined based on intensity statistics and systematic absences. The structure was solved using SHELXT (Sheldrick, 2015) and refined using full-matrix least-squares on F^2^with the OLEX^2^ suite (Dolomanov *et al.*, 2009). An intrinsic phasing solution was calculated, which provided most non-hydrogen atoms from the E-map. Full-matrix least squares / difference Fourier cycles were performed, which located the remaining non-hydrogen atoms. All non-hydrogen atoms were refined with anisotropic displacement parameters.

### Fractional atomic coordinates and isotropic or equivalent isotropic displacement parameters (Å^2^)

**Table:**

	*x*	*y*	*z*	*U*_iso_*/*U*_eq_	
S1	0.33052 (3)	0.51497 (7)	0.84291 (6)	0.0314 (3)	
F3	0.28584 (8)	0.47946 (19)	0.98737 (17)	0.0591 (8)	
O2	0.36630 (8)	0.55684 (18)	0.89278 (18)	0.0381 (7)	
O3	0.33701 (8)	0.42598 (18)	0.83416 (18)	0.0382 (7)	
N4	0.41363 (9)	0.56867 (19)	0.61232 (19)	0.0243 (7)	
F2	0.24174 (8)	0.4902 (2)	0.87503 (19)	0.0667 (9)	
N3	0.38087 (9)	0.69119 (19)	0.63308 (19)	0.0243 (7)	
F1	0.27011 (9)	0.6001 (2)	0.9347 (2)	0.0737 (9)	
N1	0.31506 (9)	0.7835 (2)	0.6882 (2)	0.0294 (7)	
N2	0.24087 (9)	0.7535 (2)	0.6981 (2)	0.0295 (7)	
O1	0.31616 (10)	0.5576 (2)	0.76506 (19)	0.0504 (8)	
C27	0.40732 (11)	0.6326 (2)	0.6686 (2)	0.0240 (8)	
H27	0.420079	0.635150	0.725618	0.029*	
C26	0.38945 (11)	0.5878 (2)	0.5366 (2)	0.0261 (8)	
H26	0.387282	0.554329	0.485583	0.031*	
C6	0.38221 (12)	0.7486 (2)	0.8345 (2)	0.0281 (8)	
H6	0.356319	0.711763	0.829601	0.034*	
C28	0.44454 (11)	0.4982 (2)	0.6233 (2)	0.0239 (8)	
C25	0.36961 (11)	0.6633 (2)	0.5499 (2)	0.0275 (8)	
H25	0.350952	0.692778	0.508910	0.033*	
C30	0.45687 (12)	0.3513 (2)	0.6343 (2)	0.0292 (8)	
H30	0.444921	0.295880	0.636712	0.035*	
C15	0.28330 (11)	0.7240 (3)	0.6763 (2)	0.0288 (9)	
H15	0.289568	0.669035	0.655722	0.035*	
C33	0.49270 (11)	0.5138 (2)	0.6266 (2)	0.0257 (8)	
C29	0.42563 (12)	0.4176 (2)	0.6266 (2)	0.0270 (8)	
C1	0.39593 (11)	0.7953 (2)	0.7613 (2)	0.0273 (8)	
C16	0.19747 (11)	0.7084 (2)	0.6921 (2)	0.0293 (9)	
C2	0.43327 (11)	0.8508 (2)	0.7742 (2)	0.0284 (8)	
H2	0.443153	0.884817	0.726861	0.034*	
C32	0.52197 (11)	0.4447 (3)	0.6345 (2)	0.0291 (9)	
H32	0.554723	0.453509	0.637309	0.035*	
C21	0.17238 (12)	0.6943 (2)	0.7690 (2)	0.0310 (9)	
C36	0.51244 (12)	0.6001 (2)	0.6162 (3)	0.0307 (9)	
H36A	0.499383	0.626166	0.563814	0.046*	
H36B	0.546454	0.596740	0.610783	0.046*	
H36C	0.504378	0.633941	0.667323	0.046*	
C5	0.40513 (12)	0.7545 (3)	0.9134 (3)	0.0326 (9)	
H5	0.395486	0.720892	0.961213	0.039*	
C7	0.38224 (12)	0.8477 (2)	0.5930 (2)	0.0295 (9)	
C31	0.50495 (12)	0.3631 (3)	0.6386 (2)	0.0306 (9)	
C17	0.18141 (12)	0.6840 (3)	0.6106 (2)	0.0299 (9)	
C12	0.42784 (12)	0.8482 (3)	0.5586 (2)	0.0321 (9)	
H12	0.450052	0.809883	0.581303	0.038*	
C19	0.11264 (12)	0.6264 (3)	0.6814 (3)	0.0331 (9)	
C20	0.12978 (13)	0.6533 (3)	0.7608 (3)	0.0344 (9)	
H20	0.111768	0.643484	0.811775	0.041*	
C34	0.37388 (11)	0.4017 (3)	0.6219 (3)	0.0329 (9)	
H34A	0.357322	0.444354	0.655747	0.049*	
H34B	0.366979	0.346176	0.645772	0.049*	
H34C	0.363669	0.404164	0.561004	0.049*	
C13	0.29209 (12)	0.8530 (3)	0.7202 (3)	0.0340 (9)	
H13	0.306240	0.904756	0.735551	0.041*	
C14	0.24595 (12)	0.8351 (3)	0.7261 (3)	0.0361 (10)	
H14	0.221897	0.871463	0.745661	0.043*	
C3	0.45621 (12)	0.8580 (3)	0.8530 (3)	0.0338 (9)	
H3	0.481451	0.896091	0.859329	0.041*	
C8	0.35079 (13)	0.9032 (3)	0.5542 (3)	0.0349 (9)	
H8	0.319328	0.903991	0.573675	0.042*	
C11	0.44155 (14)	0.9023 (3)	0.4933 (2)	0.0374 (10)	
H11	0.472803	0.901466	0.472694	0.045*	
C10	0.40973 (14)	0.9575 (3)	0.4579 (3)	0.0406 (10)	
H10	0.419101	0.995255	0.413444	0.049*	
C22	0.20862 (12)	0.7009 (3)	0.5280 (2)	0.0386 (10)	
H22A	0.219943	0.758818	0.528540	0.058*	
H22B	0.188329	0.692459	0.477424	0.058*	
H22C	0.235230	0.662562	0.524665	0.058*	
C18	0.13875 (12)	0.6426 (3)	0.6070 (3)	0.0329 (9)	
H18	0.127120	0.624903	0.552073	0.039*	
C4	0.44202 (12)	0.8092 (3)	0.9228 (3)	0.0356 (10)	
H4	0.457721	0.813342	0.977122	0.043*	
C37	0.28007 (13)	0.5219 (3)	0.9132 (3)	0.0418 (11)	
C9	0.36438 (15)	0.9577 (3)	0.4874 (3)	0.0425 (11)	
H9	0.342258	0.994901	0.462425	0.051*	
C23	0.06599 (13)	0.5824 (3)	0.6753 (3)	0.0444 (11)	
H23A	0.056297	0.564060	0.733422	0.067*	
H23B	0.068812	0.533700	0.636871	0.067*	
H23C	0.042650	0.621045	0.651571	0.067*	
C35	0.53809 (14)	0.2904 (3)	0.6448 (3)	0.0426 (11)	
H35A	0.524844	0.247568	0.683221	0.064*	
H35B	0.567944	0.309684	0.668652	0.064*	
H35C	0.543085	0.266699	0.586731	0.064*	
C24	0.18976 (15)	0.7225 (3)	0.8564 (3)	0.0419 (11)	
H24A	0.223609	0.714367	0.859655	0.063*	
H24B	0.174540	0.689721	0.902275	0.063*	
H24C	0.182489	0.781963	0.864474	0.063*	
B1	0.36969 (13)	0.7823 (3)	0.6694 (3)	0.0273 (10)	

### Atomic displacement parameters (Å^2^)

**Table:**

	*U*^11^	*U*^22^	*U*^33^	*U*^12^	*U*^13^	*U*^23^
S1	0.0263 (5)	0.0332 (6)	0.0347 (6)	−0.0018 (4)	−0.0037 (4)	0.0032 (5)
F3	0.0434 (14)	0.083 (2)	0.0510 (17)	−0.0021 (13)	0.0132 (12)	0.0086 (15)
O2	0.0316 (13)	0.0371 (18)	0.0456 (17)	−0.0116 (12)	−0.0116 (12)	0.0105 (14)
O3	0.0362 (14)	0.0332 (18)	0.0453 (18)	0.0009 (12)	−0.0041 (12)	−0.0057 (14)
N4	0.0203 (13)	0.0241 (18)	0.0284 (17)	−0.0011 (12)	0.0002 (12)	−0.0022 (14)
F2	0.0227 (12)	0.104 (3)	0.074 (2)	−0.0032 (13)	−0.0044 (12)	−0.0208 (17)
N3	0.0224 (13)	0.0230 (18)	0.0274 (16)	0.0024 (12)	−0.0004 (12)	−0.0012 (13)
F1	0.0653 (17)	0.062 (2)	0.093 (2)	0.0289 (15)	−0.0020 (16)	−0.0247 (18)
N1	0.0237 (15)	0.032 (2)	0.0327 (18)	0.0071 (13)	0.0000 (13)	−0.0064 (15)
N2	0.0241 (14)	0.035 (2)	0.0296 (17)	0.0062 (13)	−0.0014 (12)	−0.0038 (15)
O1	0.0512 (17)	0.056 (2)	0.0436 (18)	0.0009 (15)	−0.0141 (14)	0.0211 (16)
C27	0.0231 (16)	0.025 (2)	0.0240 (19)	0.0029 (14)	−0.0004 (14)	−0.0023 (16)
C26	0.0218 (16)	0.031 (2)	0.0260 (19)	0.0007 (14)	−0.0050 (14)	−0.0003 (17)
C6	0.0257 (17)	0.026 (2)	0.032 (2)	0.0032 (15)	−0.0004 (15)	0.0009 (17)
C28	0.0225 (16)	0.023 (2)	0.0264 (19)	0.0016 (14)	0.0017 (14)	0.0023 (16)
C25	0.0245 (17)	0.031 (2)	0.027 (2)	0.0037 (15)	−0.0036 (14)	−0.0006 (17)
C30	0.0357 (19)	0.022 (2)	0.030 (2)	0.0009 (16)	0.0035 (16)	−0.0021 (17)
C15	0.0232 (17)	0.029 (2)	0.034 (2)	0.0039 (15)	0.0004 (15)	−0.0005 (17)
C33	0.0276 (17)	0.026 (2)	0.0239 (19)	0.0010 (15)	0.0032 (15)	0.0006 (16)
C29	0.0301 (17)	0.029 (2)	0.0215 (18)	0.0014 (15)	0.0007 (15)	−0.0025 (16)
C1	0.0220 (16)	0.031 (2)	0.029 (2)	0.0064 (15)	0.0007 (14)	−0.0009 (17)
C16	0.0220 (17)	0.031 (2)	0.035 (2)	0.0079 (15)	−0.0015 (15)	−0.0008 (18)
C2	0.0299 (18)	0.028 (2)	0.027 (2)	−0.0007 (15)	0.0064 (15)	−0.0011 (17)
C32	0.0225 (17)	0.039 (3)	0.026 (2)	0.0063 (16)	0.0024 (14)	0.0012 (17)
C21	0.0345 (19)	0.030 (2)	0.028 (2)	0.0065 (16)	0.0003 (16)	0.0022 (18)
C36	0.0255 (17)	0.031 (2)	0.036 (2)	−0.0025 (16)	0.0002 (16)	−0.0020 (18)
C5	0.0328 (19)	0.034 (3)	0.030 (2)	0.0057 (17)	0.0022 (16)	−0.0023 (18)
C7	0.0296 (18)	0.030 (2)	0.029 (2)	0.0014 (16)	−0.0075 (15)	−0.0033 (17)
C31	0.0338 (19)	0.034 (3)	0.0237 (19)	0.0104 (17)	0.0036 (15)	−0.0027 (17)
C17	0.0259 (17)	0.034 (2)	0.030 (2)	0.0058 (16)	0.0019 (15)	0.0011 (18)
C12	0.0360 (19)	0.030 (2)	0.031 (2)	−0.0008 (16)	−0.0001 (16)	−0.0005 (18)
C19	0.0269 (18)	0.030 (2)	0.042 (2)	0.0043 (16)	0.0011 (16)	0.0012 (19)
C20	0.036 (2)	0.031 (2)	0.037 (2)	0.0047 (17)	0.0059 (17)	0.0034 (19)
C34	0.0302 (19)	0.031 (2)	0.038 (2)	−0.0092 (16)	0.0029 (16)	0.0004 (19)
C13	0.0305 (19)	0.028 (2)	0.044 (2)	0.0026 (16)	0.0009 (17)	−0.0080 (19)
C14	0.0243 (18)	0.041 (3)	0.043 (2)	0.0059 (16)	−0.0004 (16)	−0.010 (2)
C3	0.0292 (18)	0.035 (3)	0.038 (2)	−0.0032 (16)	0.0025 (16)	−0.0080 (19)
C8	0.041 (2)	0.030 (3)	0.033 (2)	0.0017 (17)	−0.0090 (17)	−0.0038 (19)
C11	0.047 (2)	0.040 (3)	0.025 (2)	−0.0025 (19)	0.0048 (17)	0.0006 (19)
C10	0.059 (3)	0.029 (3)	0.034 (2)	−0.011 (2)	−0.005 (2)	0.002 (2)
C22	0.0320 (19)	0.054 (3)	0.030 (2)	−0.0007 (18)	0.0011 (16)	−0.005 (2)
C18	0.0287 (18)	0.036 (3)	0.034 (2)	0.0064 (16)	−0.0025 (16)	−0.0002 (19)
C4	0.0278 (19)	0.048 (3)	0.031 (2)	0.0030 (17)	−0.0006 (16)	−0.006 (2)
C37	0.030 (2)	0.048 (3)	0.047 (3)	0.0011 (19)	−0.0069 (18)	−0.005 (2)
C9	0.053 (3)	0.032 (3)	0.042 (3)	0.0027 (19)	−0.016 (2)	0.006 (2)
C23	0.036 (2)	0.038 (3)	0.059 (3)	−0.0036 (18)	0.003 (2)	0.001 (2)
C35	0.042 (2)	0.041 (3)	0.045 (3)	0.0127 (19)	0.0011 (19)	−0.006 (2)
C24	0.058 (3)	0.041 (3)	0.027 (2)	0.000 (2)	0.0003 (19)	0.0040 (19)
B1	0.0191 (18)	0.024 (3)	0.038 (3)	0.0029 (16)	−0.0023 (17)	0.000 (2)

### Geometric parameters (Å, º)

**Table:**

S1—O2	1.444 (3)	C36—H36B	0.9800
S1—O3	1.440 (3)	C36—H36C	0.9800
S1—O1	1.436 (3)	C5—H5	0.9500
S1—C37	1.808 (4)	C5—C4	1.379 (5)
F3—C37	1.335 (5)	C7—C12	1.410 (5)
N4—C27	1.350 (4)	C7—C8	1.398 (5)
N4—C26	1.387 (4)	C7—B1	1.611 (6)
N4—C28	1.443 (4)	C31—C35	1.503 (5)
F2—C37	1.344 (4)	C17—C22	1.513 (5)
N3—C27	1.322 (4)	C17—C18	1.391 (5)
N3—C25	1.391 (4)	C12—H12	0.9500
N3—B1	1.592 (5)	C12—C11	1.381 (5)
F1—C37	1.323 (5)	C19—C20	1.384 (5)
N1—C15	1.330 (5)	C19—C18	1.389 (5)
N1—C13	1.382 (5)	C19—C23	1.513 (5)
N1—B1	1.592 (5)	C20—H20	0.9500
N2—C15	1.347 (4)	C34—H34A	0.9800
N2—C16	1.441 (5)	C34—H34B	0.9800
N2—C14	1.380 (5)	C34—H34C	0.9800
C27—H27	0.9500	C13—H13	0.9500
C26—H26	0.9500	C13—C14	1.356 (5)
C26—C25	1.350 (5)	C14—H14	0.9500
C6—H6	0.9500	C3—H3	0.9500
C6—C1	1.406 (5)	C3—C4	1.387 (5)
C6—C5	1.381 (5)	C8—H8	0.9500
C28—C33	1.404 (5)	C8—C9	1.401 (6)
C28—C29	1.398 (5)	C11—H11	0.9500
C25—H25	0.9500	C11—C10	1.380 (6)
C30—H30	0.9500	C10—H10	0.9500
C30—C29	1.392 (5)	C10—C9	1.376 (6)
C30—C31	1.392 (5)	C22—H22A	0.9800
C15—H15	0.9500	C22—H22B	0.9800
C33—C32	1.392 (5)	C22—H22C	0.9800
C33—C36	1.500 (5)	C18—H18	0.9500
C29—C34	1.507 (5)	C4—H4	0.9500
C1—C2	1.405 (5)	C9—H9	0.9500
C1—B1	1.611 (5)	C23—H23A	0.9800
C16—C21	1.400 (5)	C23—H23B	0.9800
C16—C17	1.390 (5)	C23—H23C	0.9800
C2—H2	0.9500	C35—H35A	0.9800
C2—C3	1.382 (5)	C35—H35B	0.9800
C32—H32	0.9500	C35—H35C	0.9800
C32—C31	1.394 (5)	C24—H24A	0.9800
C21—C20	1.391 (5)	C24—H24B	0.9800
C21—C24	1.502 (5)	C24—H24C	0.9800
C36—H36A	0.9800		
			
O2—S1—C37	102.90 (18)	C7—C12—H12	118.6
O3—S1—O2	114.52 (16)	C11—C12—C7	122.7 (4)
O3—S1—C37	102.7 (2)	C11—C12—H12	118.6
O1—S1—O2	115.16 (18)	C20—C19—C18	118.4 (3)
O1—S1—O3	115.30 (19)	C20—C19—C23	120.8 (4)
O1—S1—C37	103.82 (19)	C18—C19—C23	120.8 (4)
C27—N4—C26	107.6 (3)	C21—C20—H20	118.8
C27—N4—C28	126.7 (3)	C19—C20—C21	122.5 (4)
C26—N4—C28	125.2 (3)	C19—C20—H20	118.8
C27—N3—C25	106.6 (3)	C29—C34—H34A	109.5
C27—N3—B1	128.2 (3)	C29—C34—H34B	109.5
C25—N3—B1	124.6 (3)	C29—C34—H34C	109.5
C15—N1—C13	107.4 (3)	H34A—C34—H34B	109.5
C15—N1—B1	129.8 (3)	H34A—C34—H34C	109.5
C13—N1—B1	122.9 (3)	H34B—C34—H34C	109.5
C15—N2—C16	126.0 (3)	N1—C13—H13	125.7
C15—N2—C14	108.3 (3)	C14—C13—N1	108.6 (4)
C14—N2—C16	125.7 (3)	C14—C13—H13	125.7
N4—C27—H27	124.8	N2—C14—H14	126.8
N3—C27—N4	110.4 (3)	C13—C14—N2	106.4 (3)
N3—C27—H27	124.8	C13—C14—H14	126.8
N4—C26—H26	126.9	C2—C3—H3	120.3
C25—C26—N4	106.3 (3)	C2—C3—C4	119.4 (4)
C25—C26—H26	126.9	C4—C3—H3	120.3
C1—C6—H6	118.9	C7—C8—H8	119.1
C5—C6—H6	118.9	C7—C8—C9	121.8 (4)
C5—C6—C1	122.2 (3)	C9—C8—H8	119.1
C33—C28—N4	118.0 (3)	C12—C11—H11	120.1
C29—C28—N4	119.0 (3)	C10—C11—C12	119.8 (4)
C29—C28—C33	122.9 (3)	C10—C11—H11	120.1
N3—C25—H25	125.5	C11—C10—H10	120.1
C26—C25—N3	109.1 (3)	C9—C10—C11	119.8 (4)
C26—C25—H25	125.5	C9—C10—H10	120.1
C29—C30—H30	118.7	C17—C22—H22A	109.5
C31—C30—H30	118.7	C17—C22—H22B	109.5
C31—C30—C29	122.5 (4)	C17—C22—H22C	109.5
N1—C15—N2	109.4 (3)	H22A—C22—H22B	109.5
N1—C15—H15	125.3	H22A—C22—H22C	109.5
N2—C15—H15	125.3	H22B—C22—H22C	109.5
C28—C33—C36	122.0 (3)	C17—C18—H18	119.0
C32—C33—C28	117.1 (3)	C19—C18—C17	122.0 (4)
C32—C33—C36	120.8 (3)	C19—C18—H18	119.0
C28—C29—C34	122.4 (3)	C5—C4—C3	120.1 (4)
C30—C29—C28	117.1 (3)	C5—C4—H4	120.0
C30—C29—C34	120.6 (4)	C3—C4—H4	120.0
C6—C1—B1	120.1 (3)	F3—C37—S1	112.3 (3)
C2—C1—C6	115.8 (3)	F3—C37—F2	106.4 (4)
C2—C1—B1	124.1 (3)	F2—C37—S1	111.7 (3)
C21—C16—N2	118.1 (3)	F1—C37—S1	112.3 (3)
C17—C16—N2	119.0 (3)	F1—C37—F3	107.1 (4)
C17—C16—C21	122.9 (3)	F1—C37—F2	106.7 (3)
C1—C2—H2	118.7	C8—C9—H9	119.9
C3—C2—C1	122.6 (4)	C10—C9—C8	120.1 (4)
C3—C2—H2	118.7	C10—C9—H9	119.9
C33—C32—H32	118.8	C19—C23—H23A	109.5
C33—C32—C31	122.3 (3)	C19—C23—H23B	109.5
C31—C32—H32	118.8	C19—C23—H23C	109.5
C16—C21—C24	122.3 (3)	H23A—C23—H23B	109.5
C20—C21—C16	116.8 (3)	H23A—C23—H23C	109.5
C20—C21—C24	120.9 (3)	H23B—C23—H23C	109.5
C33—C36—H36A	109.5	C31—C35—H35A	109.5
C33—C36—H36B	109.5	C31—C35—H35B	109.5
C33—C36—H36C	109.5	C31—C35—H35C	109.5
H36A—C36—H36B	109.5	H35A—C35—H35B	109.5
H36A—C36—H36C	109.5	H35A—C35—H35C	109.5
H36B—C36—H36C	109.5	H35B—C35—H35C	109.5
C6—C5—H5	120.0	C21—C24—H24A	109.5
C4—C5—C6	120.0 (4)	C21—C24—H24B	109.5
C4—C5—H5	120.0	C21—C24—H24C	109.5
C12—C7—B1	119.0 (3)	H24A—C24—H24B	109.5
C8—C7—C12	115.7 (4)	H24A—C24—H24C	109.5
C8—C7—B1	125.3 (3)	H24B—C24—H24C	109.5
C30—C31—C32	118.1 (3)	N3—B1—C1	109.3 (3)
C30—C31—C35	121.6 (4)	N3—B1—C7	107.0 (3)
C32—C31—C35	120.3 (3)	N1—B1—N3	105.8 (3)
C16—C17—C22	122.2 (3)	N1—B1—C1	107.4 (3)
C16—C17—C18	117.4 (3)	N1—B1—C7	110.1 (3)
C18—C17—C22	120.4 (3)	C7—B1—C1	116.7 (3)
			
O2—S1—C37—F3	−64.4 (3)	C29—C30—C31—C32	−0.3 (5)
O2—S1—C37—F2	176.1 (3)	C29—C30—C31—C35	−178.6 (3)
O2—S1—C37—F1	56.3 (3)	C1—C6—C5—C4	1.7 (6)
O3—S1—C37—F3	54.8 (3)	C1—C2—C3—C4	−0.3 (6)
O3—S1—C37—F2	−64.6 (4)	C16—N2—C15—N1	178.4 (3)
O3—S1—C37—F1	175.5 (3)	C16—N2—C14—C13	−179.0 (3)
N4—C26—C25—N3	−0.4 (4)	C16—C21—C20—C19	0.8 (6)
N4—C28—C33—C32	−178.1 (3)	C16—C17—C18—C19	0.5 (6)
N4—C28—C33—C36	−2.0 (5)	C2—C1—B1—N3	111.7 (4)
N4—C28—C29—C30	177.9 (3)	C2—C1—B1—N1	−134.0 (3)
N4—C28—C29—C34	−1.9 (5)	C2—C1—B1—C7	−9.8 (5)
N1—C13—C14—N2	0.5 (4)	C2—C3—C4—C5	−0.5 (6)
N2—C16—C21—C20	177.6 (3)	C21—C16—C17—C22	179.0 (4)
N2—C16—C21—C24	−1.5 (5)	C21—C16—C17—C18	−1.0 (6)
N2—C16—C17—C22	1.8 (5)	C36—C33—C32—C31	−175.8 (3)
N2—C16—C17—C18	−178.2 (3)	C5—C6—C1—C2	−2.4 (5)
O1—S1—C37—F3	175.2 (3)	C5—C6—C1—B1	176.7 (3)
O1—S1—C37—F2	55.8 (4)	C7—C12—C11—C10	−1.3 (6)
O1—S1—C37—F1	−64.1 (3)	C7—C8—C9—C10	0.2 (6)
C27—N4—C26—C25	0.5 (4)	C31—C30—C29—C28	0.0 (5)
C27—N4—C28—C33	−64.4 (5)	C31—C30—C29—C34	179.8 (3)
C27—N4—C28—C29	118.2 (4)	C17—C16—C21—C20	0.4 (6)
C27—N3—C25—C26	0.2 (4)	C17—C16—C21—C24	−178.8 (4)
C27—N3—B1—N1	−115.8 (4)	C12—C7—C8—C9	−2.0 (6)
C27—N3—B1—C1	−0.5 (5)	C12—C7—B1—N3	−57.2 (4)
C27—N3—B1—C7	126.8 (3)	C12—C7—B1—N1	−171.7 (3)
C26—N4—C27—N3	−0.3 (4)	C12—C7—B1—C1	65.5 (5)
C26—N4—C28—C33	107.0 (4)	C12—C11—C10—C9	−0.7 (6)
C26—N4—C28—C29	−70.4 (4)	C20—C19—C18—C17	0.6 (6)
C6—C1—C2—C3	1.7 (5)	C13—N1—C15—N2	1.1 (4)
C6—C1—B1—N3	−67.4 (4)	C13—N1—B1—N3	−179.6 (3)
C6—C1—B1—N1	47.0 (4)	C13—N1—B1—C1	63.8 (4)
C6—C1—B1—C7	171.1 (3)	C13—N1—B1—C7	−64.3 (4)
C6—C5—C4—C3	−0.2 (6)	C14—N2—C15—N1	−0.8 (4)
C28—N4—C27—N3	172.3 (3)	C14—N2—C16—C21	−62.5 (5)
C28—N4—C26—C25	−172.3 (3)	C14—N2—C16—C17	114.8 (4)
C28—C33—C32—C31	0.4 (5)	C8—C7—C12—C11	2.6 (6)
C25—N3—C27—N4	0.1 (4)	C8—C7—B1—N3	120.2 (4)
C25—N3—B1—N1	74.4 (4)	C8—C7—B1—N1	5.6 (5)
C25—N3—B1—C1	−170.2 (3)	C8—C7—B1—C1	−117.1 (4)
C25—N3—B1—C7	−43.0 (4)	C11—C10—C9—C8	1.2 (6)
C15—N1—C13—C14	−1.0 (4)	C22—C17—C18—C19	−179.6 (4)
C15—N1—B1—N3	−1.0 (5)	C18—C19—C20—C21	−1.3 (6)
C15—N1—B1—C1	−117.6 (4)	C23—C19—C20—C21	−179.7 (4)
C15—N1—B1—C7	114.3 (4)	C23—C19—C18—C17	179.1 (4)
C15—N2—C16—C21	118.4 (4)	C24—C21—C20—C19	180.0 (4)
C15—N2—C16—C17	−64.2 (5)	B1—N3—C27—N4	−171.2 (3)
C15—N2—C14—C13	0.2 (4)	B1—N3—C25—C26	171.9 (3)
C33—C28—C29—C30	0.6 (5)	B1—N1—C15—N2	−177.7 (3)
C33—C28—C29—C34	−179.2 (3)	B1—N1—C13—C14	177.9 (3)
C33—C32—C31—C30	0.1 (5)	B1—C1—C2—C3	−177.4 (3)
C33—C32—C31—C35	178.4 (3)	B1—C7—C12—C11	−179.8 (4)
C29—C28—C33—C32	−0.8 (5)	B1—C7—C8—C9	−179.5 (4)
C29—C28—C33—C36	175.3 (3)		

### Bond angles around the boron center in Ph_2_B(MesIm)_2_OTf.

**Table:**

	N1 B1 N3	N1 B1 C1	N3 B1 C1	C1 B1 C7
Angle Measurements (°)	105.79	107.37	109.29	116.76
